# Chronic *Psoroptes ovis* Infestation Induces Testicular Degeneration and Submandibular Gland Hypertrophy in Rabbits (*Oryctolagus cuniculus*)

**DOI:** 10.3390/vetsci13040392

**Published:** 2026-04-17

**Authors:** María Fernanda González-Chávez, Guadalupe Arjona-Jiménez, Pablo Alejandro Bochicchio, Claudia Hallal-Calleros, Iván Flores-Pérez

**Affiliations:** 1 Facultad de Ciencias Agropecuarias, Universidad Autónoma del Estado de Morelos, Av. Universidad 1001, Chamilpa, Cuernavaca 62209, Morelos, Mexico; mafergonzalezch@gmail.com (M.F.G.-C.); lupitarjona29@gmail.com (G.A.-J.); 2 Facultad de Ciencias Exactas y Naturales, Ciudad Universitaria, Pabellón II, Intendente Güiraldes 2160, Buenos Aires C1428EGA, Argentina; pablobochi@gmail.com

**Keywords:** psoroptic mange, ectoparasite, histopathology, parasitic castration, spermatogenesis

## Abstract

Background: Psoroptic mange caused by *Psoroptes ovis* is the most common dermatological disease in rabbits; despite being an ectoparasite, in male rabbits *P. ovis* alters characteristic hormone-dependent behaviors like chinning and sexual behavior, also inducing a decrease in serum testosterone. Our goal was to evaluate the effect of *P. ovis* infestation on the histomorphology of testicular and submandibular (chinning) gland tissues of infected rabbits. Methods: Glands were obtained from experimentally infested and healthy rabbits, processed for inclusion in paraffin and stained with hematoxylin-eosin. Results: Testicular degeneration and submandibular gland hypertrophy were observed. In the testes of infested rabbits, lumen area and perimeter, seminiferous tubule area, tubule perimeter and diameter, increased. Conversely, germinal epithelium thickness and Johnsen scores of spermatogenic scale decreased, indicating impaired spermatogenesis. In the submandibular gland, lumen area and perimeter, and the total duct area and perimeter were increased. In addition, the area and perimeter of the acini also increased, while the number of acini decreased, consistent with glandular hypertrophy. Conclusions: The acarosis markedly alters the morphometric characteristics of both tissues, highlighting that psoroptic mange exerts a systemic effect on reproduction of the host altering non-dermal tissues with key roles in male reproductive function and chemical communication.

## Simple Summary

Rabbit meat is high in protein, vitamins and minerals, and low in saturated fat, cholesterol and sodium, making it an ideal food for athletes, children, elderly and people with cardiovascular diseases, and is recommended by nutritionists as part of a balanced diet. Rabbits are frequently affected by an ear mite called *Psoroptes ovis*, which causes psoroptic mange. This parasite does not infect healthy humans, but in rabbits, it causes scabs and inflammation, altering their behavior and reproductive capacity, thus impacting their well-being and producing less meat. The purpose of this study was to analyze two organs important for communication and reproduction in rabbits: the testes and the glands located in the jaw. To achieve this, we compared healthy rabbits with rabbits infected with this parasite, finding that, even though the parasite is external to the rabbit, it can induce changes in the cells of the two organs studied, since in infested rabbits, changes in the shape and organization of their cells were observed. Thus, we confirmed that, despite being a dermatological disease, it causes invisible damage that affects communication and reproduction in rabbits.

## 1. Introduction

Parasites can disrupt host physiology and behavior, including structural and functional alterations of the gonads [1,2,3,4,5]. When parasitosis impairs reproductive functions through gonadal damage or dysfunction, the phenomenon is known as parasitic castration [6]. Experimental infections with *Taenia crassiceps* cysticerci illustrate this process, since male BALB/c mice exhibit decreased androgen levels, altered sexual behavior, and marked histopathological disruption of the seminiferous epithelium [7,8], whereas in females, infection impairs hormonal profiles, reproductive performance and tissue damage in the reproductive system [9]. Among ectoparasites, *Sarcoptes scabiei* has been shown to induce systemic alterations beyond the skin, since moderate infestations in male Iberian wild goats reduce testicular mass [10], while in females, mange disrupts folliculogenesis and ovulatory capacity [11]. The domestic rabbit (*Oryctolagus cuniculus*) relies heavily on chemical communication mediated by several specialized glands. The submandibular (chinning) gland, in particular, exhibits pronounced sexual dimorphism, steroid dependence, and behavioral relevance, with secretions that modulate social dominance, sexual receptivity, and reproductive success [12,13,14,15]. These traits, however, can be vulnerable to disturbance by parasitic infections. Recent studies show that *Psoroptes ovis* (*P. ovis)*, the most prevalent ectoparasite of rabbits worldwide [16,17,18,19], not only causes dermatological lesions but also reduces locomotion, exploratory behavior, chinning frequency, feed intake, body weight, and reproductive output in both sexes [20,21]. In bucks, infestation decreases serum testosterone, prolongs reaction time to mating, and reduces mating success [21]. Although *P. ovis* is a skin parasite, indirect lesions in internal organs, including liver, spleen, lung, and intestines, have been described, suggesting a systemic impact [22]; however, these reports are descriptive and do not address whether tissues with reproductive or communicative functions are affected. Despite previous reports describing systemic effects of *Psoroptes* infestation, there is a lack of quantitative morphometric analyses specifically addressing androgen-dependent tissues involved in reproduction and chemical communication. This gap is critical, because alterations in testicular architecture can compromise spermatogenesis, and modifications in the submandibular gland may disrupt chemical communication and sexual signaling, two pillars of rabbit reproductive biology. Based on the well-documented networks that connect the immune, nervous, and endocrine systems, we hypothesized that chronic *Psoroptes* infestation induces systemic alterations resulting in measurable structural changes in androgen-dependent tissues involved in reproduction and chemical communication. Therefore, the aim of this study was to characterize, through quantitative morphometry and histopathology, the alterations induced by chronic *P. ovis* infestation in the testes and submandibular glands of male rabbits.

## 2. Materials and Methods

### 2.1. Animals and Tissues

No animals were sacrificed specifically for this study. Testicular and submandibular gland tissues were obtained from male rabbits experimentally infested with *P. ovis* that were previously used in a separate experiment [21]. The histological analyses in this manuscript are based on data from six rabbits, of which three were healthy and three were infected with a medium degree of infestation [23]; gland weight data comes from 11 healthy and 12 infested rabbits. Briefly, twenty-three clinically healthy New Zealand White male rabbits (3.2 ± 0.1 kg) individually housed in standard cages, with ad libitum access to water and a pelleted diet (Ganador^®^, Malta Cleyton, Mexico City, Mexico), were randomly assigned to a control (n = 11) or infested (n = 12) group. The infested group received 250 *P. ovis* mites into the left auditory canal, covered with cotton and adhesive tape, and removed 7 days later; 28 days after, a second inoculation with 150 mites was administered into the right ear. Mites were obtained from naturally infested rabbits and identified using stereomicroscopy according to Bowman (2011) [24] and Sanders et al. (2000) [25]. Control animals underwent the same handling procedures without mite exposure. On day 177 post-infestation, rabbits were sedated with xylazine/ketamine (5/35 mg/kg) intramuscularly and euthanized with 100 mg/kg of sodium pentobarbital, and glands were dissected.

### 2.2. Determination of Sample Size

The number of microscopic fields required for quantitative analysis was determined using G*Power 3.1 software, based on expected effect size and variance from pilot measurements. Twenty histological sections of each gland were analyzed in each rabbit (60 sections of each gland). For the analysis of testes, on each histological section eight microscopic fields were analyzed. One photomicrograph from each field was obtained (a total of 480 microphotographs) and subdivided into four quadrants. One quadrant (the lower left) was randomly selected for analysis. For the analysis of the acini of the submandibular glands, two photomicrographs per histological section were analyzed (120 photomicrographs in total), and for the striated ducts three photomicrographs per section (a total of 180). For an expected effect size ≥1.5 and α = 0.05, three animals per group yield a statistical power (1–β ≥ 0.80). This analysis incorporated effect sizes previously observed in morphometric parameters of rabbit testicular and salivary tissues in parasite challenge studies.

### 2.3. Tissue Processing

For histological analysis, samples of the left testes and left submandibular gland were collected from three healthy and three *P. ovis* chronically infested male rabbits with a medium degree of infestation [21]. Tissues were fixed in 4% paraformaldehyde for 48 h and processed using standard paraffin embedding techniques. From each tissue block, ten non-consecutive (semi-serial) longitudinal and ten transverse sections (6–8 μm thick) were obtained and stained with hematoxylin and eosin, having 20 observational units in total. Histological sections were examined using a light microscope equipped with a digital imaging system (Motic^®^) and analyzed with Motic Images Plus software 3.0. Morphometric measurements obtained from multiple non-consecutive histological sections and independent microscopic fields per animal, allows minimizing repeated sampling of the same cellular structures. These measurements represent technical replicates within each individual; therefore, values were averaged per animal, which was considered the experimental unit for statistical analysis.

### 2.4. Morphometric Analysis of Testes

The lower left quadrant was chosen randomly as the observation quadrant. All the seminiferous tubules presenting a transverse (circular) profile within this quadrant were analyzed in each observational unit ensuring unbiased sampling [26,27]; tubules extending beyond this quadrant were excluded to ensure measurement consistency. For each analyzed tubule, the parameters recorded were horizontal and vertical diameters, total area and perimeter, thickness of the germinal epithelium (mean of four equidistant measurements), and lumen area and perimeter of the tubular lumen [26,27]. Spermatogenesis was evaluated using the Johnsen scoring system (1–10 scale), following the methodological criteria described in Bolat et al. [28] which evaluate the presence, organization, and maturation of germ cells and allow identification of degenerative or necrotic changes.

To assess Leydig cell morphology, eight fields per section were examined at 40× magnification. In each field, three Leydig cells were randomly selected, and their area and perimeter were measured [26].

### 2.5. Morphometric Analysis of Submandibular Chinning Glands

Two fields per histological section were photographed at 40× magnification. Each photomicrograph was subdivided into twelve sequentially numbered quadrants (63 × 63 µm); quadrant 7 was randomly chosen for quantitative analysis. All fully visible acini within this quadrant were counted and acinar number, area (µm^2^), and perimeter (µm) were recorded [14,29].

For striated ducts, three photomicrographs at 40× magnification were obtained per section. Only ducts in transverse sections were included, and total duct area and perimeter, lumen area and perimeter, horizontal and vertical diameters, and height (length) of the epithelial cells were measured.

### 2.6. Total and Relative Weight of Glands

Rabbits were weighted with a scale (Torrey^®^ L-PCR Series, Monterrey, Mexico) before sacrifice; immediately after sacrifice, testes and submandibular glands were weighted with an analytical balance (Citizen^®^, Mexico City, Mexico) and absolute weight was recorded. Relative weight was obtained with respect to the total body weight of the rabbit, using the sum of the weight of both glands [30].

### 2.7. Statistical Analysis

Data distribution was assessed using the Kolmogorov–Smirnov test, and the D’Agostino-Pearson omnibus K^2^ test was applied to selected variables for confirmation. Variables with normal distribution (seminiferous tubule lumen perimeter, germinal epithelium thickness, epithelial cell height of striated ducts) were analyzed using Student’s t-test. Non-normal variables (body weight, total and relative weight of glands, seminiferous tubule morphometry, Johnsen scores, Leydig cell measurements, acinar morphometry, and ductal morphometry) were analyzed with the Mann–Whitney U test. Differences were considered statistically significant at *p* ≤ 0.05. All analyses were conducted using GraphPad Prism 8.0 (GraphPad Software, San Diego, CA, USA).

## 3. Results

### 3.1. Effect of P. ovis Infestation on the Morphometry of Testicular and Submandibular Glands

**Figure 1:**
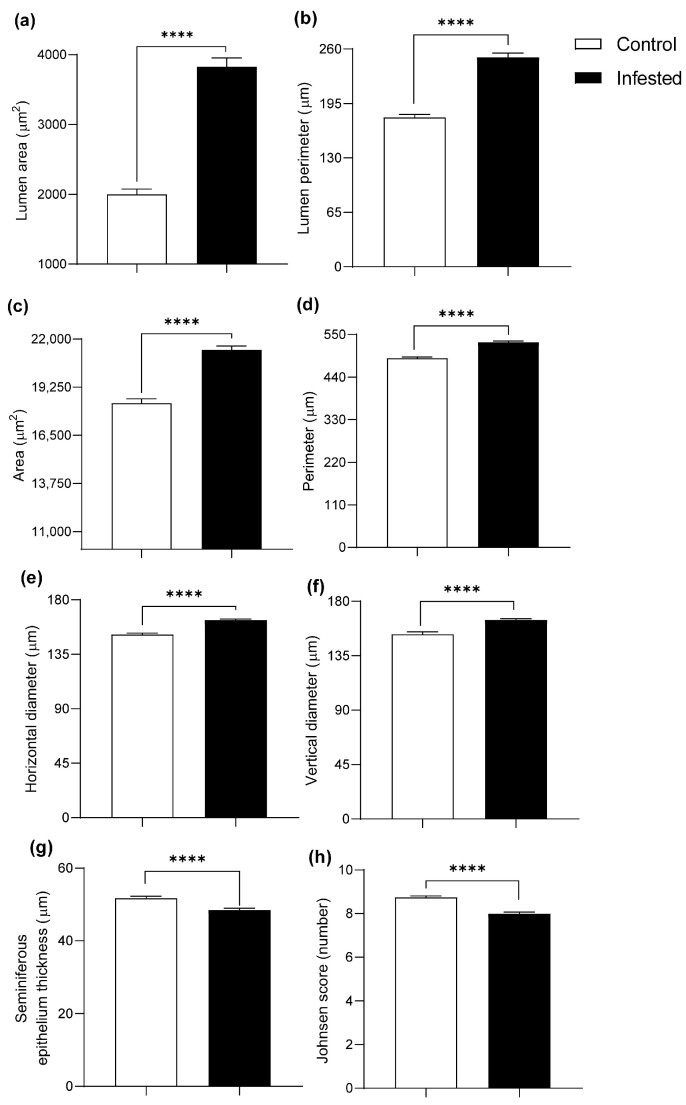
Histometry of the testis of healthy and infested male rabbits at 177 days post-infestation with *Psoroptes ovis*. Lumen (**a**) area and (**b**) perimeter. Seminiferous tubule area (**c**), perimeter (**d**), horizontal diameter (**e**), and vertical diameter (**f**). Germinal epithelium thickness (**g**) and Johnsen score (**h**). H&E stain, Mean ± SEM; (**** *p* < 0.0001).

**Figure 2:**
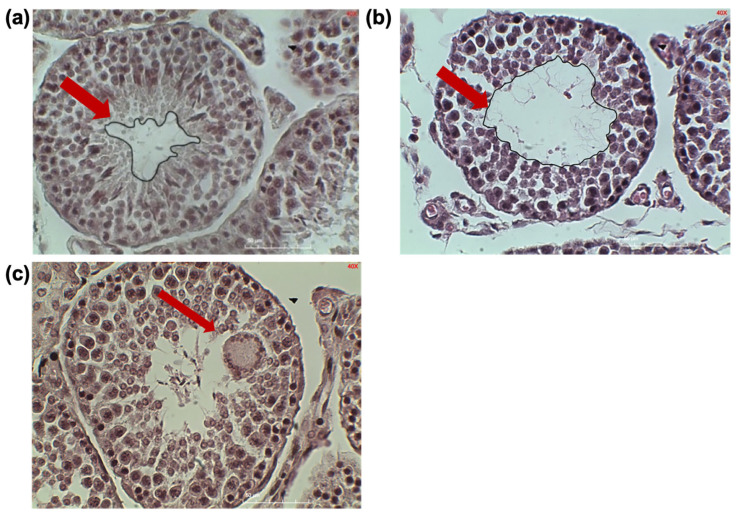
Representative images of seminiferous tubules from control (**a**) and infested (**b**) rabbits and giant cell ((**c**), arrow) in an infested rabbit.

Morphometric analysis detected pronounced changes in the testes of infested animals. Seminiferous tubules revealed a marked increase in tubule size in the infested group, with an increase in lumen area of 91.87% and lumen perimeter of 40.31% (*p* ≤ 0.0001) (Figure 1a,b and Figure 2a,b). The total area and perimeter of the tubule increased by 16.62% and 8.46%, respectively (*p* ≤ 0.0001) (Figure 1c,d and Figure 2a,b), while horizontal and vertical diameters increased by 7.74% and 7.86% (*p* ≤ 0.0001) (Figure 1e,f and Figure 2a,b).

### 3.2. Effect of Infestation on Spermatogenesis

Conversely, germinal epithelium thickness decreased by 6.25% and Johnsen scores by 8.58% in the infested group compared to the control (*p* ≤ 0.0001) (Figure 1g,h).

Occasional structural abnormalities were observed in seminiferous tubules of infested rabbits, including enlarged multinucleated cells consistent with giant cell formation (Figure 2c). Although initially described as vacuolated structures, their morphology (coalesced cytoplasm and multiple nuclei) indicates that they are spermatogenic giant cells rather than true vacuoles. In the seminiferous tubule, a slight alteration in the architecture of the germinal epithelium, along with disorganization of the cell layers and decrease in epithelial thickness, resulting in a slightly enlarged tubular lumen (Figure 2b). Sperm can be identified in the lumen, indicating the presence of spermatogenesis, but the cells are not arranged in an orderly manner, resulting in hypospermatogenesis.

### 3.3. Leydig Cell Morphology

**Figure 3:**
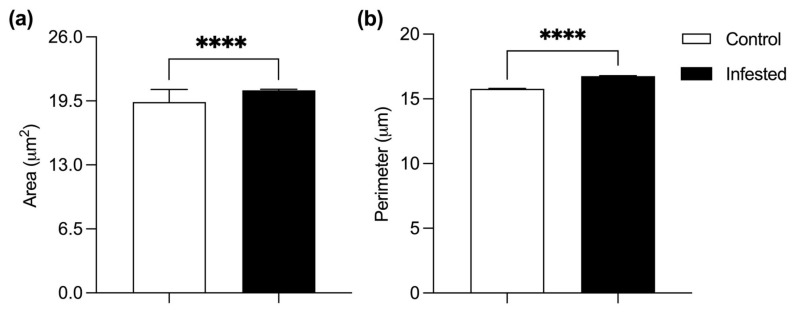
Histometry of Leydig cells in healthy or infested male rabbits at 177 days post-infestation with *Psoroptes ovis*. Cell area (**a**) and perimeter (**b**). Mean ± SEM; (**** *p* < 0.0001).

Leydig cells from infested rabbits showed a 6.14% increase in mean cell area and a 6.21% increase in perimeter compared to the control group. (*p* ≤ 0.0001) (Figure 3). This hypertrophy is compatible with a compensatory response to impaired steroidogenic function associated with chronic inflammation.

### 3.4. Submandibular Gland Morphometry

**Figure 4:**
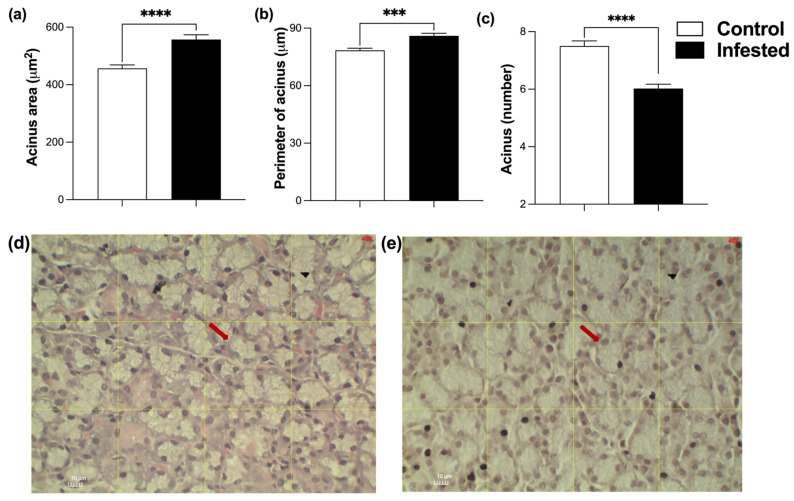
Histometry of acini from the submandibular gland of healthy and infested male rabbits at 177 days post-infestation with *Psoroptes ovis*. Acini area (**a**) and perimeter (**b**). Acini number per field (**c**). Mean ± SEM; (*** *p* ≤ 0.001, **** *p* < 0.0001). Representative images of control (**d**) and infested (**e**) rabbits; arrows indicate the acini evaluated.

Acini of infested rabbits increased a 21.97% in area (*p* ≤ 0.0001) and a 9.58% in perimeter compared to controls (*p* ≤ 0.001) (Figure 4a,b,d,e). Consistent with their larger size, the number of acini per field was significantly lower in the infested group indicating hypertrophy of secretory units (*p* ≤ 0.0001) (Figure 4c). In representative histological images, the arrows highlight acinar enlargement and dilation of luminal spaces characteristic of infested animals (Figure 4d,e).

**Figure 5:**
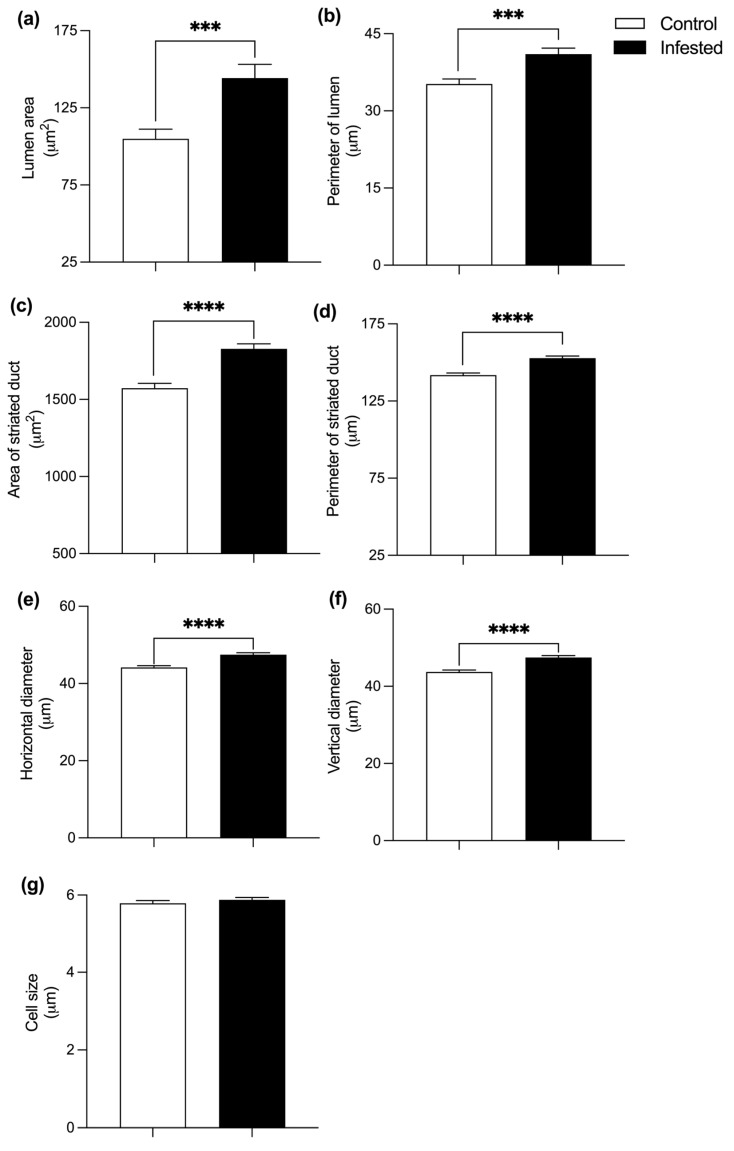
Histometry of the striated duct of the submandibular gland of healthy and infested rabbits, at 177 days post-infestation with Psoroptes ovis. Lumen area (**a**) and perimeter (**b**). Striated duct area (**c**), perimeter (**d**), horizontal diameter (**e**), vertical diameter (**f**), and cell length (**g**). Mean ± SEM; (*** *p* ≤ 0.001; **** *p* < 0.0001).

Striated ducts of infested rabbits demonstrated substantial expansion; lumen area increased by 37.5%, lumen perimeter by 16.47%, total duct area by 16.2%, and duct perimeter by 7.75% (*p* ≤ 0.0001) (Figure 5a–d). Horizontal and vertical diameters increased by 7.7% and 8.7%, respectively (Figure 5e,f). On the contrary, no differences were found in the height of the cells within the striated ducts (*p* ≥ 0.05) (Figure 5g), suggesting that ductal hypertrophy primarily reflects luminal expansion rather than epithelial remodeling.

### 3.5. Total and Relative Weight of Glands

To assess if the histomorphological alterations reported here could be attributed to variations in somatic growth, an analysis of the absolute weights and the weight of the glands in relation to the body weight of rabbits was performed. The weights of the rabbits that participated in the behavioral study (twelve infested rabbits and eleven healthy ones), from which the tissues for this study were obtained, were analyzed [21]. No significant differences were observed in either the absolute or relative weights of the testes or submandibular glands between healthy and infested rabbits (Table 1).

## 4. Discussion

The current study demonstrates that chronic *Psoroptes ovis* infestation induces significant histomorphological alterations in both the testes and the submandibular glands of male rabbits. These changes occurred despite the absence of relevant differences in growth performance, indicating that the parasite exerts systemic effect that extend beyond the skin and compromise internal organs with reproductive and communicative functions.

Testicular changes reflected a clear disruption of spermatogenesis. The marked enlargement of the seminiferous tubule lumen, reduced germinal epithelium thickness and lower Johnsen scores, suggests impaired progression of germ cells and premature sloughing of the seminiferous epithelium. Multinucleated giant spermatogenic cells, as observed in the infested group, are typically associated with germ-cell degeneration and have been described in parasitic infections capable of inducing partial or complete parasitic castration [31]. Similar lesions have been reported in murine cysticercosis caused by *Taenia crassiceps*, in which chronic infection impairs spermatogenesis and reduces testosterone levels [7,8]. Our findings are therefore consistent with the concept that chronic parasitism can compromise male fertility by altering testicular architecture and function, but they also expand the importance of ectoparasite infections in these disorders.

Leydig cells hypertrophy observed in infested rabbits may represent a compensatory response to impaired steroidogenesis. Enlarged Leydig cells have been associated with chronic inflammatory signaling, altered luteinizing hormone stimulation, or local cytokine imbalances in several models of testicular dysfunction [32,33], and proinflammatory mediators such as IL-1β and TNF-α can suppress steroid hormone synthesis even when Leydig cells appear hypertrophic or hyperplastic on histological examination [33]. This interpretation of our findings is compatible with previous behavioral and endocrine data in *P. ovis* infested bucks, which show reduced mating activity and decreased serum testosterone levels [21].

Changes in the submandibular gland further support a systemic impact of infestation. The rabbit submandibular gland is an androgen-dependent, sexually dimorphic structure whose morphology and secretory activity are tightly regulated by gonadal steroids [13,14,15]. Thus, acinar hypertrophy together with a reduced number of acini and enlargement of striated ducts documented in the current work, suggest alterations in hormone composition or secretory dynamics that could impair chemical communication. These morphometric changes provide a plausible anatomical substrate for the previously documented reduction in chinning behavior and social/sexual signaling in infested males [21]. Unfortunately, in this work we did not measure the chemical components of these glands, which limits our understanding of the observed histological alterations.

Importantly, the morphological differences reported here cannot be attributed to body growth or nutritional status. In a separate study using the same animals, no significant differences were observed between control and infested rabbits in body weight, zoometric index, voluntary feed intake, or routine physiological markers [21]. Thus, the lesions described in testicular and submandibular tissues represent true pathological consequences of *P. ovis* infestation rather than artifacts of somatic development or malnutrition.

The mechanisms underlying these alterations likely involve interactions between systemic inflammation, endocrine disruption, and energetic reallocation. Chronic irritation and otitis caused by *P. ovis* can trigger local and systemic inflammatory responses, including cytokine and acute-phase protein production [34,35]. Persistent stress associated with mange are also capable of dysregulating the hypothalamic–pituitary–gonadal axis, leading to reduced gonadotropin secretion and impaired steroidogenesis [36]. In addition, transcriptomic and immunological studies in psoroptic mange and related ectoparasitoses have demonstrated activation of TLR-dependent pathways and NF-κB signaling, which can mimic the endocrine and testicular effects reported for systemic lipopolysaccharide exposure [37,38]. Together, these pathways offer a coherent explanation for the concurrent degeneration of seminiferous epithelium, Leydig cell hypertrophy, and remodeling of submandibular gland architecture observed in the present study.

Although the sample size seems relatively small, a priori power analysis indicated that three animals per group were sufficient to detect large, biologically meaningful effect sizes in morphometric variables having enough observation units of each tissue. These findings reflect biologically relevant effects detected with adequate statistical power; however, they should be interpreted within the context of the experimental design and the specific conditions of this study.

Another limitation of this study is that we did not obtain biochemical data with which we could correlate and support our observations. In future works, it would be desirable to quantify hormonal (cortisol, LH, FSH) or cytokine profiles, as well as to assess oxidative stress markers, sperm quality, or fertility outcomes. Such measurements would help to clarify the causal pathways linking *P. ovis* infestation to reproductive dysfunction and to validate the concept of parasitic castration more directly in this model. Thus, future studies integrating morphological, endocrine, immunological, and reproductive performance data will be essential to fully elucidate the biological impact of this highly prevalent ectoparasite.

## 5. Conclusions

Chronic *P. ovis* infestation elicits substantial histomorphological alterations in androgen-dependent tissues of male rabbits, including testicular degeneration and submandibular gland hypertrophy, independent of somatic growth performance. These findings highlight the importance of the systemic impact of psoroptic mange and provide mechanistic support for understanding the observed impairment in male reproductive behavior. The precise mechanisms by which *P. ovis* activates the immune and endocrine responses of its host remain unknown and warrant further investigation.

## Figures and Tables

**Table 1:** Weight of submandibular glands and testes of healthy or infested rabbits with *P. ovis* at day 177 post-infestation.

	Control	Infested	*p* Value
Left submandibular gland (g)	0.87 ± 0.13	0.82 ± 0.04	0.34
Right submandibular gland (g)	0.83 ± 0.05	0.83 ± 0.04	0.97
Relative submandibular glands weight (%)	0.04 ± 0.00	0.04 ± 0.00	0.90
Left testis (g)	5.47 ± 0.48	5.13 ± 0.66	0.49
Right testis (g)	5.16 ± 0.43	5.48 ± 0.70	0.89
Relative testes weight (%)	0.27 ± 0.02	0.28 ± 0.03	0.84

Values are expressed as mean ± SEM; Mann–Whitney test.

## Data Availability

The original contributions presented in this study are included in the article/Supplementary Materials. Further inquiries can be directed to the corresponding authors.

## Supplementary Materials

The following supporting information can be downloaded at: https://www.mdpi.com/article/10.3390/vetsci13040392/s1.
